# 

**Published:** 1918-06-08

**Authors:** 


### CONTENTS.

PAGE

**The Economic Relations of Functional Nervous Diseases** ... ... ... ... ... 195

**An Indian Civil Medical Service** ... ... ... ... 196

**Hospital and Institutional News** ... ... ... 197

American Wounded at Windsor—The New Extensions at Leeds—Another Hospital Amalgamation?—The Bombing of Hospitals—Birthday Honours—King George's Fund for Sailors—The After Treatment of Wounded—An American Surgeon on Ireland—Bacilli and Business—Hospital Visitation in France—A New Mechano-Therapeutical Department—The New Auxiliary War Hospitals—Women Out-patients and the Mineral Water Hospital, Bath—The Standardisation of Salaries—The Remuneration of Registrars—Treatment of London Consumptives—Asylum Workers' Association—Aberdare Hospital—This Week's Drug Market—To Our Readers.

**The Great War** ... ... ... ... ... ... 201

IX. Flies and Disease.

**War Influences on the Medical Profession** ... 202

The General Medical Council's Problems.

PAGE

**Human Temperaments** ... ... ... ... 205

The Shy Person.

**The Economics of Nursing** ... ... ... ... 207

A Nurse's Charter of Liberty: Viscount Knutsford's Reply to "Ierne."

**The Trained Nurse the Nation's Creditor** ... 207

**The Matrons' and Sisters' Department** ... 209

District Nursing: I. A Historical Sketch.

Round the Hospitals.

A Great Day for British Nurses—Edinburgh, Plymouth, and Truro Form College Centres—War Retards Recovery: The Remedy—When a Fourth Year's Training is Valuable—Australian Private Nurses' Standard Fees—A Nursing Home Hotch Potch.

**The After-Care of Disabled Men** ... ... ... 213

The Second Inter-Allied Conference.

**The Editor's Letter-Box** ... ... ... ... 214

**The London Hospital** ... ... ... ... ... 215

**The College of Nursing (Limited by Guarantee)** 215

